# Corps étranger intra urétral inhabituel chez un adolescent schizophrène: à propos d’un cas

**DOI:** 10.11604/pamj.2018.31.217.15773

**Published:** 2018-12-03

**Authors:** Mustapha Ahsaini, Mohammed Bounoual, Soufiane Mellas, Jalaleddine El Ammari, Mohammed Fadl Tazi, Mohammed Jamal El Fassi, Moulay Hassan Farih

**Affiliations:** 1 Service d’Urologie, Centre Hospitalier Universitaire Hassan II de Fès, Maroc

**Keywords:** Corps étranger, automutiltation, schizophrènie, endoscopie, chirurgie, Foreign body, automutiltation, schizophrenia, endoscopy, surgery

## Abstract

L'auto insertion d'un corps étranger s'observe généralement chez les patients ayant une psychose chronique dans le but d'automutilation ou érotique. Le diagnostic est parfois difficile s'il est rapporté tardivement ou si le patient est non coopérant, d'où l'intérêt d'un bilan d'imagerie complémentaire. Le traitement comporte deux volets: l'extraction de corps étranger soit par voie endoscopique ou chirurgie ouverte et la prise en charge psychiatrique de la maladie mentale. Nous rapportons une observation inhabituelle d'un adolescent de 16ans schizophrène qui s'est introduit une aiguille dans son urètre depuis 2ans dont le diagnostic a été fait à l'aide de l'urétrocystographie rétrograde et mictionnelle, et grâce à des artifices techniques, l'aiguille a été enlevée endoscopiquement sans avoir recours à la chirurgie ouverte malgré l'ancienneté de l'incident avec bien sûr complément de prise en charge psychiatrique.

## Abstract (en)

Self- insertion of foreign body is a common practice for self-injure or erotic purpose in patients with chronic psychosis. The diagnosis is sometimes difficult if it is reported late or if the patient does not cooperate; hence the interest of complementary imaging assessment. Treatment was based on two steps: the extraction of the foreign body by endoscopy or open surgery and psychiatric treatment of the mental illness. We report the uncommon case of a 16-year old schizophrenic adolescent who had introduced a needle into the urethra 2 years before. Diagnosis was based on retrograde and mictional uretrocystography. The needle was removed with endoscopy as well as with technical artifices, without using open surgery despite the length of time the incident had occurred. The patient underwent complementary psychiatric treatment.

## Introduction

Le corps étranger intra urétral constitue une affection rare, souvent rapporté dans la littérature sous forme des cas isolés [1-3]. La présence d'un corps étranger dans l'appareil urinaire pose toujours la question sur les circonstances d'introduction et l'état psychologique de patient. Il s'agit des situations iatrogènes suite à une intervention chirurgicale à ciel ouvert ou endoscopique où le corps étranger s'introduit dans le bas de l'appareil urinaire [1, 4]. Mais il a été décrit des cas d'introduction de corps étranger volontairement et fortuitement par le patient lui-même dans le but érotique ou chez des patients ayant des troubles psychiatriques [2, 3, 5]. Le patient se présente généralement avec une symptomatologie variable faite d'urétrorragie, syndrome irritatif vésical, mais parfois pas de symptômes évidents d'où l'intérêt d'un interrogatoire bien conduit [1, 2]. Le traitement est le plus souvent endoscopique [1, 2, 5]. A travers le cas d'un adolescent connu schizophrène ayant introduit dans son urètre une aiguille de coudre et une revue de la littérature, nous allons déterminer la nature de ces objets, leurs modalités d'introduction, le profil psychologique des malades, leur diagnostic et les particularités thérapeutiques. L'intérêt de notre travail, en plus de la rareté de l'affection, réside sur le jeune âge et l'ancienneté de l'incident ainsi que les particularités thérapeutiques.

## Patient et observation

**Figure 1:**
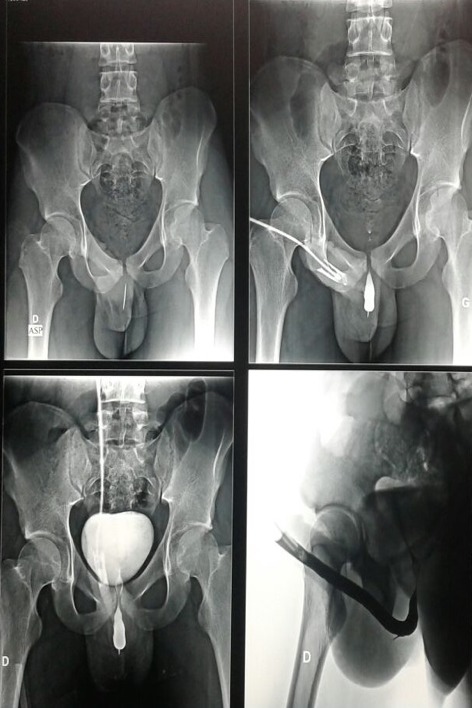
urétrocystographie rétrograde montrant un corps étranger en projection de l’urètre bulbo membraneux avec migration du bout proximal

**Figure 2:**
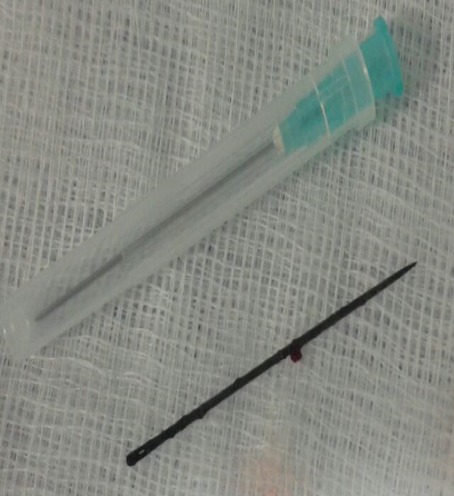
aiguille à coudre après extraction

Il s'agit d'un adolescent de 16 ans, suivi en psychiatrie pour troubles schizophréniques avec une mauvaise observance thérapeutique, qui a été amené par ses parents à la consultation urologique pour notion de corps étranger intra urétral. Le patient ne présentait pas de symptômes urinaires francs en dehors d'une simple dysurie et douleur urétrale surtout au moment de la miction. L'interrogatoire était difficile avec le patient vu les troubles délirants qu'il présente. La famille rapporte une notion d'insertion d'une aiguille de coudre par le patient lui-même il y a 2ans. L'examen clinique notamment celle de l'urètre périnéal trouve une induration avec perception d'un corps métallique sans aucuns autres signes associés. Une urétrocystographie rétrograde (UCR) (Figure 1) a été demandée montrant un corps étranger intra urétral: c'est une aiguille qui a été enclavée dans l'urètre bulbaire avec migration à peu près de 2cm du bout distal dans la paroi de l'urètre bulbaire. Une urétro-cystoscopie sous anesthésie loco régionale à la fois diagnostique et thérapeutique a été réalisée, après avoir vérifié la stérilité des urines, montrant une aiguille fine noire au niveau de l'urètre bulbo membraneux près de sphincter striés. On a pu l'extraire par voie endoscopique à l'aide d'une pince à corps étranger, en utilisant une optique 30° et en suivant l'axe de l'aiguille au moment de l'extériorisation de l'aiguille (Figure 2). Les suites opératoires ont été simples et le patient a pu quitter l'hôpital le même jour. Le patient est adressé par la suite à la consultation psychiatrique pour contrôler et traiter sa pathologie mentale. Le suivi sur le plan urologique du patient n'a pas objectivé des troubles mictionnels avec un recul de 12 mois.

## Discussion

L'insertion d'un corps étranger pose plusieurs questions sur le mode d'introduction. L'insertion iatrogène se fait accidentellement en per opératoire notamment fragilisation du matériel d'endoscopie introduit dans l'urètre: fragments de sondes, pièces défectueuses d'endoscopie [2]. L'auto insertion volontaire se produit généralement par des adultes, mais il y a des cas d'adolescents ou d'enfants rapportés dans la littérature, c'est le cas aussi de notre patient [1]. Les objets introduits sont de nature variables (aiguille, épingle, crayon, tubes, fil de pêche, câbles) [1, 2, 6]. Il s'agit souvent des patients déjà connus porteurs d'une maladie psychiatrique, mais parfois on se retrouve devant des cas sains et stables mentalement ou parfois l'incident nous amène à diagnostiquer une maladie mentale suite aux investigations psychiatriques [1, 3]. Chez notre patient, probablement l'introduction de l'aiguille dans l'urètre est faite dans un but érotique lors de masturbation pour aboutir à l'orgasme ou par curiosité sexuelle. Il a été décrit aussi dans la littérature des cas d'introduction dans le cadre des conduites d'automutilation des schizophrénies [1, 3]. Le corps étranger intra urétral ou intra vésical peut rester en dedans ou perforer et migrer dans les structures adjacentes péri-vésicales notamment le péritoine et les intestins ou les tissus péri urétraux entrainant ainsi une inflammation avec risque septique [1]. Le diagnostic peut être facile et bien porté par l'interrogatoire s'il s'agit d'un patient bien coopérant et stable sur le plan mental avec une symptomatologie évocatrice faite de douleur urétrale, périnéale ou pelvienne, urétrorragie, hématurie, urgenturie, dysurie ou rétention d'urine. On peut palper une induration douloureuse de l'urètre en regard du corps étranger surtout s'il s'agit de l'urètre antérieur [1, 5, 7]. L'arbre urinaire sans préparation (AUSP) confirme la présence d'un corps étranger radio opaque, l'UCR constitue l'examen de référence pour analyser les rapports du corps étranger par rapport aux structures adjacentes comme c'était le cas de notre patient. L'échographie a son intérêt dans les corps étrangers intra vésicaux surtout s'ils sont radio transparents à la radiographie standard. La tomodensitométrie (TDM) porte son intérêt en cas de migration dans les tissus péri vésicaux en déterminant la taille, la forme, l'emplacement et l'orientation des objets migrés [1, 4]. L'urétro-cystoscopie reste l'examen clé pour le diagnostic et dans la plupart des cas pour la thérapeutique. Dans les cas négligés ou vus tardivement le diagnostic est difficile car la symptomatologie est moins évocatrice ou parfois compliquée (nécrose septique du pénis rapporté par Hwang CE *et al.* [8], abcès scrotal rapporté par Navarro Gil J *et al.* [9]). Le pronostic dépend de la rapidité du diagnostic et de la prise en charge, ainsi les patients diagnostiqués et opérés tardivement peuvent laisser des séquelles urinaires et/ ou sexuelles [1]. L'extraction des corps étrangers urétraux ou vésicaux se fait le plus souvent par voie endoscopique à l'aide d'une pince de préhension, avec bien sûr adaptation technique, en utilisant une optique de 30° à fin de s'adapter à l'orientation du corps étranger comme le cas de notre patient [1, 2, 4, 5]. La chirurgie à ciel ouvert est surtout réservée en cas de corps étranger volumineux, ou à ceux ayant migré dans les tissus avoisinants ou après échec de l'endoscopie [5, 7]. Par la suite l'urologue est obligé d'orienter ces patients vers une consultation psychiatrique afin de dépister et de traiter une éventuelle maladie mentale notamment dépression mélancolique ou schizophrénie dans le but de prévenir tout geste d'automutilation dans le futur, ainsi de dégager sa responsabilité juridique [1, 3, 10].

## Conclusion

L'insertion d'un corps étranger dans le bas appareil urinaire se fait le plus souvent par des malades mentaux adultes connus ou diagnostiqués, rarement par des enfants et adolescents, sans méconnaitre des cas d'insertion sous l'effet de drogues ou dans un but sexuel. Le diagnostic précoce est facile en se basant sur les aveux des patients et de leur entourage, l'imagerie et surtout l'endoscopie, cette dernière a aussi un rôle thérapeutique dans la plupart des cas. Le traitement psychiatrique fait partie de la prise en charge globale de patient dans un intérêt préventif.

## Conflits d’intérêts

Les auteurs ne déclarent aucun conflit d'intérêts.
